# Regulatory Roles of Long Non-Coding RNAs in Methotrexate Pharmacology: Mechanistic and Translational Insights

**DOI:** 10.1007/s11095-026-04087-3

**Published:** 2026-04-07

**Authors:** Carlos A. Guzmán-Martín, Nadia Janet González-Moyotl, Yaneli Juárez-Vicuña, Rafael Bojalil, Laura Aline Martínez-Martínez, Ana Laura Arenas-Díaz, Martín Martínez-Rosas, Mario Peña-Peña, Miguel Angel Vázquez-Toledo, Rodrigo Romero-Nava, Rogelio Frank Jiménez-Ortega, Alberto Hidalgo-Bravo, Rafael Velázquez-Cruz, Fausto Sánchez-Muñoz

**Affiliations:** 1 Doctorado en Ciencias Biológicas y de La Salud, Universidad Autónoma Metropolitana, Ciudad de Mexico, México; 2 Departamento de Programas de Investigación, Hospital Shriners México, Ciudad de Mexico, México; 3 Departamento de Genética, Instituto Nacional de Ciencias Médicas y Nutrición Salvador Zubirán, Ciudad de Mexico, México; 4 Departamento de Inmunología, Instituto Nacional de Cardiología Ignacio Chávez, Ciudad de Mexico, México; 5 Universidad Autónoma Metropolitana, Ciudad de Mexico, México; 6 Departamento de Reumatología, Instituto Nacional de Cardiología Ignacio Chávez, Ciudad de Mexico, México; 7 Staff Médico, Hospital Shriners México, Ciudad de Mexico, México; 8 Departamento de Fisiología, Instituto Nacional de Cardiología Ignacio Chávez, Ciudad de Mexico, México; 9 Posgrado en Ciencias en Inmunología, Escuela Nacional de Ciencias Biológicas, Instituto Politécnico Nacional (ENCB-IPN), Ciudad de Mexico, México; 10 Laboratorio de Investigación en Genética de Enfermedades Metabólicas, Escuela Superior de Medicina, Instituto Politécnico Nacional, Ciudad de Mexico, México; 11 Servicio de Medicina Genómica, Instituto Nacional de Rehabilitación Luis Guillermo Ibarra Ibarra (INRLGII), Ciudad de Mexico, México; 12 Laboratorio de Genómica del Metabolismo Óseo, Instituto Nacional de Medicina Genómica (INMEGEN), Ciudad de Mexico, México

**Keywords:** Cancer, LncRNas, Long non-coding RNas, Lung adenocarcinoma, Methotrexate, Pharmacodynamics, Pharmacology, Rheumatoid arthritis

## Abstract

**Background:**

Methotrexate (MTX) remains a cornerstone therapy in autoimmune diseases and oncology; however, substantial interindividual variability in efficacy and toxicity persists. While variability in MTX pharmacokinetics (PK) and pharmacodynamics (PD) has been linked to transporters, folate-cycle enzymes, and intracellular polyglutamate accumulation, the contribution of long non-coding RNAs (lncRNAs) as regulatory modifiers of these processes is not yet systematically defined.

**Objective:**

To synthesize current evidence on lncRNA-mediated regulation of MTX response and to organize these interactions within a pharmacology-centered mechanistic framework.

**Methods:**

A narrative, mechanism-oriented review was conducted integrating preclinical, translational, and computational studies evaluating lncRNA involvement in MTX-related pathways across immune-mediated diseases and cancer.

**Results:**

lncRNAs were found to intersect with MTX pharmacology across four principal mechanistic nodes: (1) regulation of drug transport and intracellular retention (e.g., modulation of ABC transporters); (2) control of folate-axis targets and compensatory metabolic pathways (e.g., DHFR and TYMS regulation); (3) modulation of adenosine-mediated immunoregulation and NF-κB signaling, with lincRNA-p21 representing the most mechanistically supported example in rheumatoid arthritis; and (4) orchestration of stress-adaptive and survival pathways influencing antifolate resistance (e.g., PI3K-AKT-mTOR and apoptosis networks). However, most evidence derives from in vitro or computational studies, with limited integration of lncRNA perturbation and quantitative PK metrics such as intracellular MTX-polyglutamate levels.

**Conclusions:**

lncRNAs function as context-dependent modulators of MTX PK/PD processes rather than primary drug targets. Current evidence is heterogeneous, future studies integrating functional genomics, PK measurements, and prospective clinical validation are required to establish lncRNAs as predictive biomarkers or therapeutic targets in MTX precision pharmacology.

## Introduction

Methotrexate (MTX) remains a foundational therapy for autoimmune diseases and certain malignancies; however, substantial interindividual variability in efficacy and toxicity continues to limit its precise use. While high-dose methotrexate (HD-MTX) exerts cytotoxic effects primarily through inhibition of dihydrofolate reductase (DHFR) and disruption of folate-dependent nucleotide synthesis, low-dose regimens particularly in rheumatoid arthritis (RA) and psoriasis operate predominantly via anti-inflammatory mechanisms involving adenosine accumulation and suppression of NF-κB-mediated signaling [1, 2]. Across indications, variability in drug transport, intracellular retention, target engagement, and downstream signaling pathways contributes to heterogeneous clinical outcomes, underscoring the need to better define the molecular determinants of MTX pharmacokinetics (PKs) and pharmacodynamics (PDs) [3].

Long non-coding RNAs (lncRNAs), defined as transcripts longer than 200 nucleotides that lack protein-coding potential, have emerged as context-specific regulators of gene expression and cellular signaling [4–6]. Rather than serving as passive transcriptional byproducts, lncRNAs actively modulate chromatin organization, transcriptional programs, RNA stability, and signal transduction pathways. Importantly, these regulatory functions position lncRNAs as potential modulators of drug response by influencing the expression of transporters, metabolic enzymes, inflammatory mediators, and survival pathways [7–9].

Accumulating evidence suggests that lncRNAs influence MTX activity across various disease contexts. In RA, lincRNA-p21 has been implicated in mediating MTX-associated suppression of NF-κB signaling [10]. In malignancies, lncRNAs such as TUG1, HOTAIR, and KCNQ1OT1 have been linked to MTX resistance through competing endogenous RNA (ceRNA) mechanisms and regulation of apoptotic pathways [11–13]. However, current knowledge remains fragmented, with findings dispersed across distinct disease models and varying levels of experimental validation.

To address this gap, this review adopts a pharmacology-centered framework to organize lncRNA-MTX interactions into four mechanistic categories: [1] regulation of drug transport and intracellular retention; [2] modulation of the folate pathway and polyglutamation-dependent target engagement; [3] control of adenosine-NF-κB-mediated immunoregulation; and [4] orchestration of stress-adaptive and survival programs that influence therapeutic resistance. By critically synthesizing preclinical and translational evidence within established pharmacologic principles, we aim to clarify where mechanistic causality is supported, where associations remain exploratory, and how lncRNA biology may inform precision MTX therapy across immune-mediated and oncologic diseases.

## Methotrexate

MTX is a dose-dependent antifolate with well-established roles in oncology and immune-mediated diseases. Its therapeutic effects result from context-specific PD mechanisms involving drug transport, intracellular retention, target engagement, and modulation of downstream signaling pathways [1].

In oncologic settings, HD-MTX primarily acts by inhibiting DHFR, thereby impairing tetrahydrofolate regeneration and disrupting thymidylate and purine synthesis. Following cellular uptake, predominantly via solute carrier family 19 member 1 (SLC19A1), MTX undergoes polyglutamation to form methotrexate polyglutamates (MTX-PGs). These metabolites exhibit enhanced intracellular retention and increased affinity for DHFR, thymidylate synthase (TYMS), and other folate-dependent enzymes, thereby amplifying cytotoxic effects in rapidly proliferating cells [1]. Polyglutamation is a critical determinant of pharmacologic potency and represents a central mechanism linking intracellular drug exposure to therapeutic response.

At lower doses, such as those used in RA and other inflammatory disorders, MTX exerts predominantly immunomodulatory effects. Inhibition of AICAR transformylase/IMP cyclohydrolase (ATIC) leads to the accumulation of adenosine, which activates A2A and A2B receptors on immune cells, thereby suppressing pro-inflammatory cytokine production and leukocyte activation [14].

MTX has also been shown to attenuate NF-κB signaling and modulate the Janus kinase/signal transducer and activator of transcription (JAK/STAT) pathway in specific contexts, further contributing to its anti-inflammatory effects [2]. Another important mechanism involves the induction of reactive oxygen species (ROS), which triggers autophagy and apoptosis, particularly in non-target tissues such as reproductive cells. This mechanism contributes to several well-documented adverse effects of MTX, including hepatotoxicity, myelosuppression, and reproductive toxicity [3, 15].

**Fig. 1:**
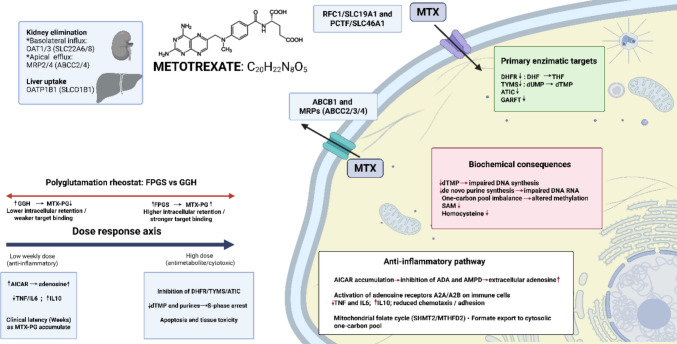
Mechanistic overview of MTX pharmacology across different dose ranges. MTX enters cells primarily via RFC1 (SLC19A1) and PCFT (SLC46A1) and is exported by ABCB1 (P-glycoprotein, MDR1) and ABCC2 (MRP2), ABCC3 (MRP3), and ABCC4 (MRP4). Intracellular MTX undergoes polyglutamation catalyzed by FPGS, which enhances its retention and affinity for key folate-dependent enzymes, including DHFR, TYMS, ATIC, and GARFT. Inhibition of these enzymes disrupts thymidylate and purine synthesis, impairs one-carbon metabolism, and alters methylation capacity. In inflammatory settings, ATIC inhibition promotes adenosine accumulation, activating A2A/A2B receptors and suppressing pro-inflammatory signaling. The balance between FPGS and GGH regulates intracellular MTX-polyglutamate levels, functioning as a rheostat for pharmacologic potency. At low weekly doses, adenosine-mediated immunomodulation predominates; at high doses, classical antimetabolite effects drive cytotoxicity. Renal (OAT1/3; MRP2/4) and hepatic (OATP1B1) transporters contribute to systemic disposition and interindividual variability in MTX exposure, therapeutic response, and toxicity

**Fig. 2:**
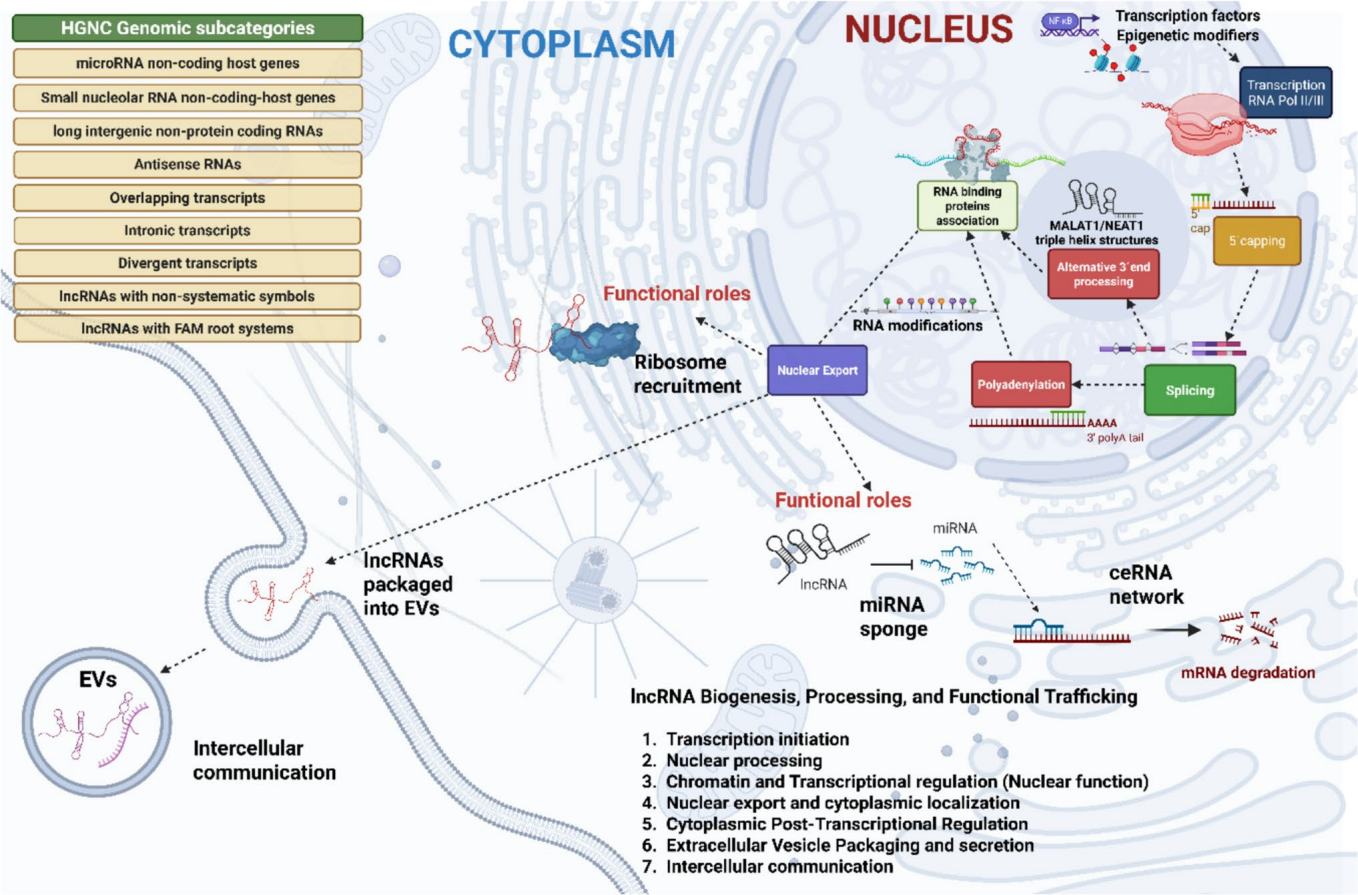
Overview of lncRNA biogenesis, processing, and functional trafficking. lncRNAs are primarily transcribed by RNA polymerase II and undergo canonical RNA processing steps, including 5′ capping, splicing, and polyadenylation. Within the nucleus, lncRNAs interact with chromatin modifiers and RNA-binding proteins to regulate transcriptional programs and nuclear architecture. Post-transcriptional modifications, such as N6-methyladenosine (m6A), influence RNA stability and subcellular localization. While a subset of lncRNAs is retained in the nucleus, others are exported to the cytoplasm, where they modulate mRNA translation and stability or function as ceRNAs by sequestering microRNAs. Certain lncRNAs are selectively packaged into extracellular vesicles (EVs) and released, enabling intercellular communication. Collectively, these processes illustrate the dynamic lifecycle of lncRNAs across nuclear, cytoplasmic, and extracellular compartments

Pharmacokinetically, MTX demonstrates variable oral bioavailability and extensive tissue distribution. Intracellular retention depends on both transporter-mediated uptake and polyglutamation efficiency, while elimination occurs primarily through renal excretion. At higher concentrations, nonlinear clearance may occur due to saturation of tubular secretion [16]. Importantly, interindividual variability in transporters, folate-cycle enzymes, and polyglutamate accumulation contributes to heterogeneous clinical responses. Polymorphisms in genes such as SLC19A1, ATIC, and TYMS, as well as differences in intracellular MTX-PG levels, have been associated with variability in efficacy and toxicity, particularly in RA [17]. Collectively, these PK and PD determinants including transport, polyglutamation, folate-axis inhibition, and adenosine-mediated immunoregulation define the molecular landscape within which regulatory factors, such as lncRNAs, may influence MTX responsiveness (Figs. 1, 2 and 3).

## Long Non-Coding RNAs (lncRNAs)

lncRNAs are transcripts longer than 200 nucleotides that lack protein-coding potential and function as regulatory molecules within gene expression networks [18]. Unlike mRNAs, lncRNAs often exhibit tissue- and context-specific expression patterns and operate through diverse mechanisms, including chromatin remodeling, transcriptional regulation, post-transcriptional control, and modulation of signal transduction pathways.

Mechanistically, lncRNAs act in cis or trans as molecular scaffolds, guides, decoys, or ceRNAs that sequester microRNAs. Through these functions, they influence the expression of transporters, metabolic enzymes, inflammatory mediators, and survival pathways-processes directly relevant to PK and PD variability. Canonical examples such as HOTAIR, Antisense Noncoding RNA in the INK4 Locus (ANRIL), Nuclear Paraspeckle Assembly Transcript 1 (NEAT1), and MALAT1 illustrate how lncRNAs can reshape chromatin states, alter transcriptional programs, or regulate RNA stability in disease contexts [19–21].

Although many lncRNAs remain incompletely characterized, their restricted tissue distribution and dynamic regulation make them attractive candidates as biomarkers and modulators of therapeutic response. Emerging technologies, including Clustered Regularly Interspaced Short Palindromic Repeats (CRISPR)-based perturbation systems, interactome mapping, and long-read sequencing, are accelerating their functional annotation. In parallel, epitranscriptomic RNA modifications, particularly N6-methyladenosine (m6A), the most abundant internal modification in eukaryotic RNA have been shown to influence lncRNA stability and protein interactions, thereby adding an additional regulatory layer to gene expression control [22].

Within this regulatory landscape, lncRNAs are increasingly recognized as potential determinants of drug responsiveness, including the modulation of MTX transport, folate-axis signaling, inflammatory pathways, and resistance mechanisms.

## LncRNAs/MTX Association in Diseases

**Fig. 3:**
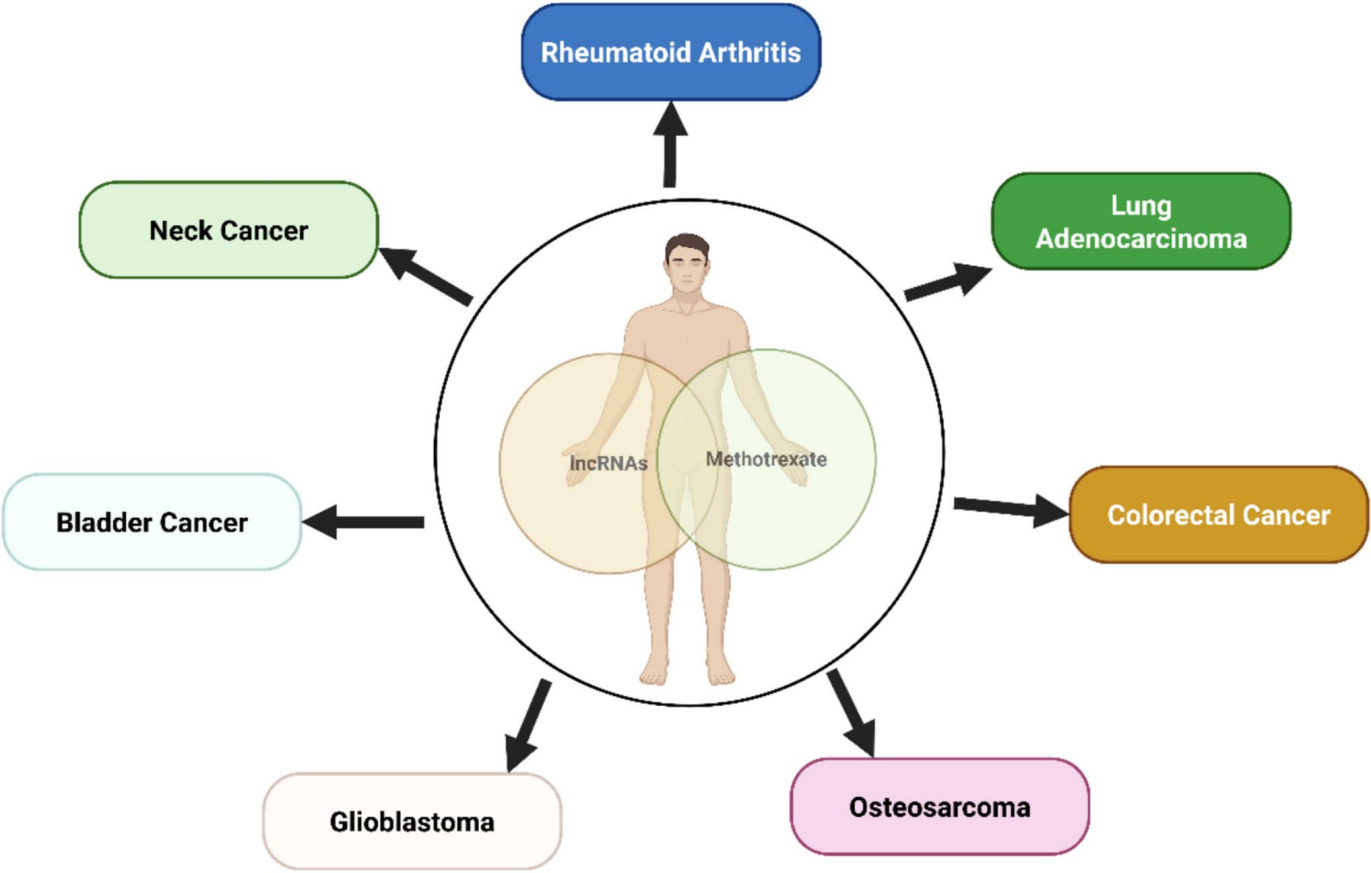
lncRNA–MTX associations across diseases. Representative conditions in which lncRNAs have been implicated in modulating methotrexate pharmacodynamics or therapeutic response. Central overlap indicates the shared regulatory network between lncRNA biology and MTX mechanisms, with disease-specific contexts shown around the periphery

### Rheumatoid Arthritis

RA is a chronic autoimmune disease characterized by synovial inflammation and progressive joint destruction. MTX remains the first-line disease-modifying antirheumatic drug (DMARD), exerting its clinical effects predominantly through adenosine-mediated immunoregulation and suppression of NF-κB–dependent inflammatory signaling [23–27]. However, variability in therapeutic response persists, suggesting that additional regulatory mechanisms remain to be defined. Among lncRNAs, lincRNA-p21 is the most mechanistically characterized candidate in RA. In untreated patients, lincRNA-p21 expression is reduced compared to healthy controls, whereas MTX exposure restores its expression via a DNA-dependent protein kinase catalytic subunit (DNA-PKcs)-dependent pathway [10]. Functional studies indicate that lincRNA-p21 contributes to the suppression of NF-κB activity, thereby reinforcing MTX-associated anti-inflammatory signaling. Importantly, these observations are derived from ex vivo patient samples and mechanistic cellular models, providing moderate support for a causal role in MTX response, although prospective clinical validation remains limited.

Beyond lincRNA-p21, evidence for additional lncRNAs influencing MTX responsiveness in RA remains fragmentary and largely associative. Although altered lncRNA expression profiles have been reported in RA synovium and peripheral blood, a direct link to MTX PD endpoints such as intracellular MTX-polyglutamate accumulation or longitudinal treatment response has not been systematically established.

Collectively, current data support lincRNA-p21 as a plausible mechanistic mediator of MTX-induced immunoregulation, while the broader contributions of lncRNAs remain exploratory. Future studies integrating lncRNA profiling with PK markers and clinical response metrics are necessary to establish their translational utility as predictive biomarkers or therapeutic targets in RA.

### Lung Adenocarcinoma

Lung adenocarcinoma (LUAD), the most common subtype of non–small-cell lung cancer, is characterized by molecular heterogeneity driven by recurrent oncogenic alterations, including mutations in the epidermal growth factor receptor (EGFR), Kirsten Rat Sarcoma Viral Oncogene Homolog (KRAS), and mesenchymal–epithelial transition (MET) factor genes [28–33]. Although MTX is not a standard therapy for LUAD, transcriptomic analyses have suggested potential interactions between lncRNA-regulated pathways and antifolate sensitivity. Several studies have identified lncRNA-based signatures associated with LUAD prognosis and autophagy regulation. A seven-lncRNA autophagy-related signature including FAM83A Antisense RNA 1(FAM83A-AS1), Long Intergenic Non-Protein Coding RNA 1116 (LINC01116), ILF3 Divergent Transcript (ILF3-DT), and Endogenous Bornavirus Like Nucleoprotein 3, Pseudogene (EBLN3P) stratifies patients by overall survival and, through connectivity mapping approaches, computationally links MTX to autophagy-associated transcriptional networks [34]. Similarly, immune-related lncRNA pair scores have been reported to correlate with tumor mutational burden and immune checkpoint expression, with in silico models predicting differential sensitivity to MTX [35].

Importantly, these findings are primarily derived from retrospective transcriptomic datasets and bioinformatic drug-repurposing analyses. Direct pharmacological validation such as MTX exposure experiments, intracellular polyglutamate quantification, or functional perturbation of candidate lncRNAs remains limited. Although mechanistic annotations implicate Forkhead box O (FoxO), AMP-activated protein kinase (AMPK), and immune regulatory pathways, causality between specific lncRNAs and MTX response in LUAD has not been experimentally established.

Collectively, current evidence positions lncRNA signatures in LUAD as hypothesis-generating frameworks rather than validated predictors of MTX responsiveness. Future studies integrating functional perturbation, PK measurements, and prospective validation will be necessary to determine whether lncRNA-defined subsets can meaningfully predict antifolate sensitivity or support rational MTX repurposing strategies in lung cancer.

### Colorectal Cancer

Colorectal cancer (CRC) is a molecularly heterogeneous malignancy driven by cumulative alterations in pathways such as wingless-related integration site (WNT)/(APC) Adenomatous Polyposis Coli, Rat Sarcoma virus, Rapidly Accelerated Fibrosarcoma, Mitogen-activated protein kinase kinase, Extracellular signal-regulated kinase (RAS–RAF–MEK–ERK), Phosphoinositide 3-kinase (PI3K), Transforming Growth Factor beta (TGF-β), and Tumor Protein p53 (TP53) [36, 37]. Although MTX has historically been used as a biochemical modulator of 5-fluorouracil (5-FU), its direct therapeutic efficacy in CRC is limited, and resistance remains a significant challenge [38, 39]. Emerging evidence suggests that lncRNA-mediated regulatory networks may contribute to antifolate resistance in preclinical CRC models.

Functional studies in CRC cell lines have identified H19, TUG1, and KCNQ1OT1 as key regulators of MTX responsiveness. H19 is upregulated in MTX-resistant HT-29 cells and promotes Wnt/β-catenin signaling, leading to increased expression of proliferative targets such as Proto-Oncogene C-Myc (c-MYC) and Cyclin D1 (CCND1). Silencing H19 restores drug sensitivity in vitro [40]. Similarly, TUG1 functions as a ceRNA for miR-186, resulting in derepression of Cytoplasmic Polyadenylation Element-Binding Protein 2 (CPEB2) and enhanced cell survival; inhibition of TUG1 re-sensitizes resistant cells [11]. KCNQ1OT1 sequesters miR-760, preventing repression of Protein Phosphatase 1 Regulatory Inhibitor Subunit 1B (PPP1R1B) and activating pro-survival cAMP response element-binding protein (cAMP/CREB) signaling. Knockdown of KCNQ1OT1 increases apoptosis and partially reverses MTX resistance in cellular models [12].

These findings provide mechanistic support for lncRNA-miRNA-mRNA circuits as contributors to MTX resistance in CRC. However, the evidence is largely restricted to in vitro systems, with validation in patient-derived samples or in vivo MTX exposure models remaining limited. Moreover, the relationship between these lncRNAs and intracellular MTX-polyglutamate accumulation or transporter activity has not been systematically examined.

Collectively, current data support a functional role for specific lncRNAs in modulating antifolate sensitivity in CRC cell models. However, their translational utility as predictive biomarkers or therapeutic targets requires prospective validation and integration with PK and clinical response parameters.

### Osteosarcoma

Osteosarcoma (OS) is a high-grade primary bone malignancy characterized by genomic instability and frequent disruption of TP53 and RB Transcriptional Corepressor 1 (RB1) signaling pathways [41–44]. Unlike several other solid tumors discussed in this review, HD-MTX with leucovorin rescue remains a core component of frontline multimodal therapy for OS [45, 46]. Nevertheless, both intrinsic and acquired resistance to MTX significantly limit long-term survival.

Preclinical studies have implicated lncRNAs as key regulators of MTX responsiveness in OS cell models. Specifically, Small Nucleolar RNA Host Gene 1 (SNHG1) and TUG1 have been reported to function as ceRNAs, sequestering tumor-suppressive microRNAs; miR-16 and miR-186, respectively, resulting in the derepression of pro-survival targets such as B-cell lymphoma 2 (BCL2) and CCND1 during MTX exposure. Knockdown of these lncRNAs partially restores chemosensitivity in vitro [47]. Similarly, EBLN3P has been shown to promote MTX resistance by sponging miR-200a-3p and upregulating O-GlcNAc transferase (OGT), thereby enhancing proliferative and epithelial-mesenchymal transition–associated pathways in cellular models.

These findings provide functional evidence supporting lncRNA-miRNA-mRNA regulatory circuits that contribute to antifolate resistance in OS. However, most data are derived from established cell lines, with limited validation in patient-derived xenografts or clinical samples exposed to HD-MTX. Moreover, direct relationships between these lncRNAs and PK factors such as MTX-polyglutamate accumulation or transporter regulation have not been systematically investigated. Overall, lncRNA-mediated ceRNA networks represent plausible contributors to MTX resistance in OS, but their integration into predictive or therapeutic frameworks will require prospective validation and correlation with pharmacologic exposure parameters in clinical cohorts.

### Glioblastoma

Glioblastoma (GBM) is a high-grade primary brain tumor characterized by marked genomic and epigenetic heterogeneity, including alterations in the EGFR, Tumor Protein 53 (TP53), Phosphatase and Tensin Homolog (PTEN), and Isocitrate Dehydrogenase 1/2 (IDH1/2) genes, as well as the methylation status of the O6-Methylguanine-DNA Methyltransferase (MGMT) promoter, which influences responsiveness to alkylating agents [48–50]. Standard treatment consists of surgical resection followed by radiotherapy and temozolomide (TMZ); MTX is not routinely used in GBM therapy.

In this context, MTX has primarily been employed in preclinical models as a pharmacologic stressor to investigate antifolate-induced transcriptional remodeling, rather than as a conventional cytotoxic agent [51]. Transcriptomic studies have identified m6A-modified lncRNAs, including AC005229.3, SRY-box transcription factor 21 antisense divergent transcript 1 (SOX21-AS1), and SWI/SNF Complex Interacting GAS6 Enhancer Non-Coding RNA (LINC00565), which correlate with glioma progression and resistance to TMZ, often associated with dysregulation of the Methyltransferase 3 (METTL3)/(YTHDF2) YTH N6-methyladenosine RNA binding protein F2 methylation axis [52]. However, these findings are largely correlative and focus on resistance to alkylating agents rather than the PDs of antifolates.

More directly relevant to MTX, HOTAIR has been shown in GBM cell models to function as a ceRNA that regulates the miR-214-3p/β-catenin/MGMT axis. In vitro studies suggest that MTX exposure reduces HOTAIR and β-catenin expression, thereby decreasing MGMT levels and partially restoring TMZ sensitivity in resistant cells [13]. These findings indicate that MTX may modulate lncRNA-driven chemoresistance networks under controlled experimental conditions.

Overall, evidence supporting a role for lncRNAs in MTX responsiveness in GBM remains limited to preclinical models, with no established clinical validation. Although antifolate-induced modulation of lncRNA programs presents a mechanistically intriguing avenue, its translational relevance in GBM requires rigorous evaluation using models that integrate drug exposure, DNA repair dynamics, and clinical outcome measures.

### Other Cancers

Beyond OS, where HD-MTX is a standard therapeutic component, evidence for most other solid tumors discussed in the literature primarily derives from transcriptomic analyses and pharmacogenomic modeling rather than from clinical studies involving antifolate exposure. In bladder cancer, a 12-lncRNA immune-infiltration–related signature stratified prognosis and was associated, through computational drug-sensitivity modeling, with lower predicted MTX IC50 values in low-risk tumors [53].

Similarly, immune-related lncRNA models in cervical squamous cell carcinoma have suggested differential predicted sensitivity to MTX across risk subgroups [54]. In head and neck squamous cell carcinoma (HNSCC), a cuproptosis-related lncRNA signature was correlated with survival and immune features, with high-risk tumors displaying lower modeled MTX IC50 values in pharmacogenomic datasets [55]. Importantly, these findings are based on retrospective transcriptomic correlations and in silico drug-response predictions rather than direct MTX exposure experiments.

Preclinical cell line studies in hepatocellular and gastrointestinal cancers have implicated lncRNAs such as H19, Cáncer urotelial asociado 1 (UCA1), and Plasmacytoma Variant Translocation 1 (PVT1) in regulating pathways relevant to antifolate stress. These pathways include ABCB1-mediated efflux, phosphatidylinositol 3-kinase–protein kinase B–mammalian target of rapamycin (PI3K–AKT–mTOR)signaling, and apoptosis regulators such as BCL2 and CCND1 [56]. However, validation of these mechanisms in vivo particularly regarding MTX polyglutamate accumulation or transporter function remains limited.

Collectively, the available data suggest that lncRNA expression profiles may intersect with immune, metabolic, and stress-adaptation pathways that influence antifolate responsiveness. However, most evidence remains computational or preclinical, and prospective validation integrating PK exposure metrics with clinical outcomes is necessary before lncRNA-based signatures can be considered predictive of MTX efficacy across these tumor types.

### Antifolate Toxicity and Stress-Response Contexts

Beyond therapeutic resistance, lncRNAs have been implicated in mediating organ-specific responses to MTX-induced stress. These studies provide insight into how antifolate exposure reshapes transcriptional programs outside oncologic contexts.

In embryonic models, transcriptomic analyses of MTX-exposed mouse embryos have identified dysregulated lncRNAs associated with neural and cardiac developmental pathways, including LINC00094, NEAT1, and MALAT1 [57, 58]. These findings are primarily descriptive and derived from global expression profiling, with limited functional validation to establish causality.

In renal toxicity models, MALAT1 has been shown to be upregulated following MTX exposure, activating the PI3K–AKT–mTOR signaling pathway and promoting tubular epithelial apoptosis in vitro. Pharmacological inhibition or knockdown of MALAT1 attenuates injury markers, suggesting a functional role in antifolate-induced nephropathy in preclinical models [59]. Similarly, hepatic injury models report increased H19 expression after MTX exposure, which correlates with NF-κB activation and inflammatory cytokine production [40]. However, these observations remain largely cell-line or animal-based.

Collectively, these data suggest that lncRNAs may play a role in cellular stress adaptation to antifolate exposure, potentially influencing organ toxicity. Whether these regulatory circuits can serve as predictive biomarkers for MTX-related adverse effects in clinical settings remains to be determined.

## Integrated Mechanistic Landscape of lncRNA–MTX Interactions

**Fig. 4:**
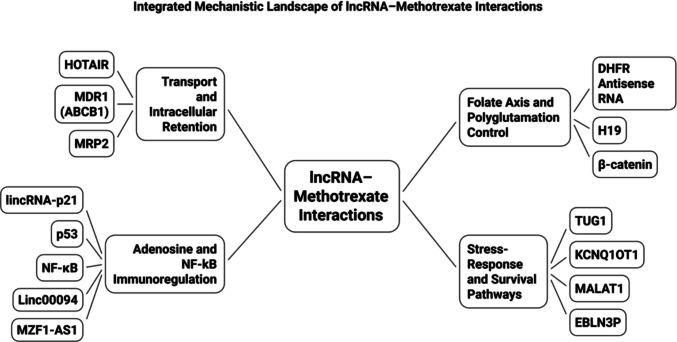
Integrated mechanistic framework of lncRNA–MTX interactions. The schematic categorizes lncRNA-MTX interactions into four primary pharmacologic nodes: [1] transport and intracellular retention, where lncRNAs (e.g., HOTAIR) regulate efflux transporters such as ABCB1 and members of the MRP family, thereby influencing intracellular MTX exposure; [2] folate-axis regulation, including DHFR antisense RNA (also referred to as DHFR2 RNA) and H19-mediated β-catenin signaling, which modulate the expression of target enzymes; [3] adenosine-NF-κB immunoregulation, exemplified by lincRNA-p21 in autoimmune contexts; and [4] stress-adaptive survival pathways, where lncRNAs such as TUG1, KCNQ1OT1, MALAT1, and EBLN3P influence apoptotic and autophagic responses under antifolate stress. Selected immune-associated lncRNAs (e.g., LINC00094 and MZF1-AS1) are depicted as modulators of inflammatory signaling

lncRNAs intersect with MTX pharmacology across distinct yet interconnected mechanistic nodes that influence intracellular drug exposure, target engagement, immunoregulation, and stress adaptation. Rather than acting through a single dominant pathway, lncRNAs modulate both PK factors such as transport and intracellular retention and PD processes, including folate-axis inhibition, inflammatory signaling, and apoptotic commitment. The framework summarized in Fig. 4 and Table 1 organizes these interactions into four functional nodes, each supported by varying degrees of experimental evidence [60, 61]. Across these nodes, most data derive from functional perturbation studies in established cell lines; direct integration with clinical MTX exposure metrics remains rare.

**Table 1:** Mechanistic Nodes Linking lncRNAs to Methotrexate Pharmacology

Mechanistic Node	Representative lncRNAs	Primary Target/Pathway	PK or PD Axis	Experimental Evidence	Translational Status
Transport and Intracellular Retention	HOTAIR	↑ ABCB1, ↑ MRP2	PK (drug efflux)	In vitro (cancer cell lines)	Exploratory; no MTX-PG integration
Folate Axis Regulation	DHFR antisense RNA (also referred to as DHFR2 RNA)	DHFR transcript stability	PD (target engagement)	In vitro	Limited clinical validation
	H19	↑ DHFR, ↑ TYMS via β-catenin	PD (compensatory enzyme expression)	In vitro (CRC models)	Preclinical
Adenosine–NF-κB Immunoregulation	lincRNA-p21	↓ NF-κB signaling; consistent with enhanced MTX-associated anti-inflammatory signaling	PD (anti-inflammatory effect)	Ex vivo RA samples + cellular models	Partial translational support
Stress Adaptation and Survival Signaling	TUG1	↑ PI3K–AKT–mTOR; miR-186 axis	PD (apoptosis resistance)	In vitro	Preclinical
	KCNQ1OT1	miR-760/PPP1R1B signaling	PD (survival signaling)	In vitro	Preclinical
	MALAT1	Autophagy modulation (BECN1, LC3)	PD (stress adaptation)	In vitro + limited in vivo	Exploratory
	EBLN3P	EMT and OGT signaling	PD (chemoresistance mechanisms)	In vitro	Exploratory
Immune/Computational Predictive Signatures	Multiple (tumor-specific panels)	Immune infiltration, autophagy, cuproptosis signatures	Predicted PD	Computational modeling	Hypothesis-generating

### Transport and Intracellular Retention

At the level of drug availability, lncRNAs influence intracellular MTX concentration by regulating membrane transporters. In cancer cell models, HOTAIR has been shown to increase the expression of the efflux transporter ABCB1 and, in some contexts, members of the MRP family. Elevated transporter expression reduces intracellular MTX accumulation and diminishes the cytotoxic response [13]. These findings are primarily supported by in vitro data demonstrating altered transporter expression and restored drug sensitivity following lncRNA silencing.

This node represents a PK-modulating axis: lncRNA expression alters intracellular exposure without directly modifying MTX’s enzymatic targets. Whether these regulatory circuits correlate with intracellular MTX-PG accumulation or clinically meaningful resistance in vivo remains to be systematically evaluated, particularly in HD-MTX settings such as OS, where polyglutamation kinetics critically determine therapeutic exposure.

### Folate Axis and Polyglutamation Control

The folate metabolism node includes the regulation of MTX target enzymes and one-carbon metabolic flux. DHFR antisense RNA (also referred to as DHFR2 RNA) acts as a cis-acting regulator of DHFR transcript stability, creating a feedback loop that may influence cellular sensitivity to antifolate drugs. Experimental models have shown that changes in antisense RNA expression alter DHFR levels and affect MTX responsiveness; however, clinical validation remains limited.

In parallel, H19 has been linked to β-catenin–dependent transcriptional upregulation of DHFR and TYMS in CRC models, constituting a compensatory mechanism that may buffer antifolate-induced stress. These findings suggest that lncRNAs can indirectly raise the threshold required for effective enzyme inhibition.

Notably, direct regulation of MTX polyglutamation enzymes (FPGS or GGH) by lncRNAs has not yet been conclusively demonstrated, representing a key mechanistic gap in the current literature.

### Adenosine–NF-κB Immunoregulatory Axis

In autoimmune contexts, MTX exerts its primary therapeutic effect through intracellular accumulation of AICAR, enhanced extracellular adenosine signaling, and suppression of NF-κB–driven inflammatory pathways. LincRNA-p21 is the most mechanistically substantiated lncRNA within this axis. In RA models and patient-derived cells, MTX has been shown to induce lincRNA-p21 expression via a DNA-PKcs–dependent mechanism, resulting in reduced NF-κB transcriptional activity and downregulation of pro-inflammatory target genes [10]. These findings provide direct experimental evidence that antifolate exposure can modulate lncRNA-mediated inflammatory signaling.

This node is supported by both mechanistic studies and analyses of patient-derived samples; however, prospective longitudinal validation as a predictive biomarker remains limited. Compared to oncologic contexts, this axis currently represents one of the most translationally grounded lncRNA-MTX interactions.

### Stress-Response and Survival Pathways

In neoplastic models exposed to MTX, lncRNAs frequently regulate adaptive stress responses that determine apoptotic versus cytostatic outcomes. TUG1 and KCNQ1OT1 maintain PI3K-AKT-mTOR signaling under antifolate pressure through ceRNA-mediated derepression of pro-survival targets. Additionally, MALAT1 and EBLN3P have been implicated in modulating autophagy and apoptosis through the regulation of BECN1, LC3, and BCL2-related pathways.

These interactions are largely supported by cell line experiments and, in some cases, xenograft models. They reflect PD-modulating effects, as lncRNA networks alter cellular susceptibility to antifolate-induced apoptosis without necessarily changing intracellular MTX levels.

The table categorizes representative lncRNAs into four principal pharmacologic nodes: transport and intracellular retention, folate-axis regulation, adenosine-NF-κB immunoregulation, and stress adaptation and survival signaling. It highlights their primary molecular targets, classification within PK or PD axes, and the current level of experimental support. The strength of evidence reflects the highest level of validation reported in the literature and does not imply consistent replication across different disease contexts. Evidence categories are defined as follows: In vitro functional perturbation studies in established cell lines; Ex vivo analyses performed in patient-derived tissues or primary cells; In vivo animal or xenograft models with controlled drug exposure; computational transcriptomic, bioinformatic, or pharmacogenomic modeling without direct MTX exposure experiments.

## Conclusions

MTX remains a cornerstone therapy for immune-mediated diseases and certain malignancies; however, variability in its efficacy and toxicity limits its precision in clinical application. Emerging evidence indicates that lncRNAs modulate MTX response across four principal mechanistic nodes: transport and intracellular retention, folate-axis regulation, adenosine-NF-κB immunomodulation, and stress-adaptive survival signaling. Within these pathways, lncRNAs act as regulatory modifiers that influence established PK and PD processes rather than serving as primary drug targets.

The evidentiary landscape is heterogeneous. Immune-regulatory mechanisms, particularly those involving lincRNA-p21, have partial translational support in RA. In contrast, transporter regulation, folate-axis compensation, and survival pathway modulation are primarily supported by in vitro and xenograft models. Computational lncRNA signatures predicting antifolate sensitivity in solid tumors remain hypothesis-generating and require experimental validation under defined MTX exposure conditions.

Critical knowledge gaps persist. Few studies have integrated lncRNA perturbation with quantitative PK metrics, such as intracellular MTX polyglutamate accumulation. Prospective clinical validation is limited, and direct regulation of polyglutamation enzymes FPGS and GGH by lncRNAs has yet to be demonstrated. Addressing these gaps will require coordinated PK/PD modeling approaches, functional genomic perturbation in patient-derived systems, and longitudinal biomarker studies.

Framing lncRNAs within established pharmacological principles provides a structured foundation for refining MTX precision therapy and outlines the experimental steps necessary for clinical translation.

## References

[1] Cronstein BN. THE MECHANISM OF ACTION OF METHOTREXATE. Vol. 23. 1997. Report. 10.1016/s0889-857x(05)70358-6 9361153

[2] Thomas S, Fisher KH, Snowden JA, Danson SJ, Brown S, Zeidler MP. Methotrexate is a JAK/STAT pathway inhibitor. PLoS One. 2015. 10.1371/journal.pone.0130078. 26131691 10.1371/journal.pone.0130078 PMC4489434

[3] Hamed KM, Dighriri IM, Baomar AF, Alharthy BT, Alenazi FE, Alali GH, et al. Overview of methotrexate toxicity: a comprehensive literature review. Cureus. 2022;14(9):e29518. 10.7759/CUREUS.29518. 36312688 10.7759/cureus.29518 PMC9595261

[4] González-Ramírez J, Leija-Montoya AG, Serafín-Higuera N, Guzmán-Martín CA, Amezcua-Guerra LM, Olvera-Sandoval C, et al. Increased Expression of lncRNA AC000120.7 and SENP3-EIF4A1 in Patients with Acute Respiratory Distress Syndrome Induced by SARS-CoV-2 Infection: A Pilot Study. Microorganisms. 2023;11(9):2342. 10.3390/MICROORGANISMS11092342. 37764186 10.3390/microorganisms11092342 PMC10537196

[5] Guzmán-Martín CA, Juárez-Vicuña Y, Domínguez-López A, González-Ramírez J, Amezcua-Guerra LM, Martínez-Martínez LA, et al. LncRNAs dysregulation in monocytes from primary antiphospholipid syndrome patients: a bioinformatic and an experimental proof-of-concept approach. Mol Biol Rep. 2022(1). 10.1007/S11033-022-08080-Y. 10.1007/s11033-022-08080-y 36367661

[6] Policarpo R, Sierksma A, De Strooper B, d’Ydewalle C. From junk to function: LncRNAs in CNS health and disease. Front Mol Neurosci. 2021;14:714768. 10.3389/FNMOL.2021.714768/XML. 34349622 10.3389/fnmol.2021.714768 PMC8327212

[7] Prabhakar B, Zhong XB, Rasmussen TP. Exploiting Long Noncoding RNAs as Pharmacological Targets to Modulate Epigenetic Diseases. Yale J Biol Med. 2017Mar 1;90(1):73 (**PubMed PMID: 28356895**). 28356895 PMC5369047

[8] Ning B, Yu AM. RNA therapeutics: from biochemical pharmacology to technology development and clinical applications. Biochem Pharmacol. 2021;189:114567. 10.1016/J.BCP.2021.114567. 33865832 10.1016/j.bcp.2021.114567 PMC9514225

[9] Juárez-Vicuña Y, Ruiz-Ojeda D, González-Ramírez J, Flores-Balderas X, Springall R, Sánchez-Muñoz F, et al. LncRNA MALAT1 in keratinocyte function: a review of recent advances. Noncoding RNA Res. 2024;9(2):594. 10.1016/J.NCRNA.2024.01.021. 38532797 10.1016/j.ncrna.2024.01.021 PMC10963180

[10] Spurlock CF, Tossberg JT, Matlock BK, Olsen NJ, Aune TM. Methotrexate inhibits NF-κB activity via lincRNA-p21 induction. Arthritis Rheumatol. 2014;66(11):2947. 10.1002/ART.38805. 25077978 10.1002/art.38805 PMC4211976

[11] Li C, Gao Y, Li Y, Ding D. TUG1 mediates methotrexate resistance in colorectal cancer via miR-186/CPEB2 axis. Biochem Biophys Res Commun. 2017;491(2):552–7. 10.1016/j.bbrc.2017.03.042. 28302487 10.1016/j.bbrc.2017.03.042

[12] Xian D, Zhao Y. LncRNA KCNQ1OT1 enhanced the methotrexate resistance of colorectal cancer cells by regulating miR-760/PPP1R1B via the cAMP signalling pathway. J Cell Mol Med. 2019;23(6):3808–23. 10.1111/JCMM.14071. 30997746 10.1111/jcmm.14071 PMC6533496

[13] Lan T, Quan W, Yu DH, Chen X, Wang ZF, Li ZQ. High expression of LncRNA HOTAIR is a risk factor for temozolomide resistance in glioblastoma via activation of the miR-214/β-catenin/MGMT pathway. Sci Rep. 2024. 10.1038/s41598-024-77348-z. 39482401 10.1038/s41598-024-77348-z PMC11528118

[14] Friedman B, Cronstein B. Methotrexate mechanism in treatment of rheumatoid arthritis. Joint Bone Spine. 2019(3). 10.1016/j.jbspin.2018.07.004. 10.1016/j.jbspin.2018.07.004 PMC6360124 30081197

[15] Xiong S, Song D, Xiang Y, Li Y, Zhong Y, Li H, et al. Reactive oxygen species, not Ca2+, mediates methotrexate-induced autophagy and apoptosis in spermatocyte cell line. Basic Clin Pharmacol Toxicol. 2020;126(2):144–52. 10.1111/bcpt.13306. 10.1111/bcpt.13306 31420979

[16] Inoue K, Yuasa H. Molecular basis for pharmacokinetics and pharmacodynamics of methotrexate in rheumatoid arthritis therapy. Drug Metab Pharmacokinet. 2014;29(1):12–9. 10.2133/DMPK.DMPK-13-RV-119. 24284432 10.2133/dmpk.dmpk-13-rv-119

[17] Dervieux T, Furst D, Lein DO, Capps R, Smith K, Walsh M, et al. Polyglutamation of methotrexate with common polymorphisms in reduced folate carrier, aminoimidazole carboxamide ribonucleotide transformylase, and thymidylate synthase are associated with methotrexate effects in rheumatoid arthritis. Arthritis Rheum. 2004;50(9):2766–74. 10.1002/art.20460. 15457444 10.1002/art.20460

[18] Mattick JS, Amaral PP, Carninci P, Carpenter S, Chang HY, Chen LL, et al. Long non-coding RNAs: definitions, functions, challenges and recommendations. Nat Rev Mol Cell Biol. 2023;24(6):430–47. 10.1038/s41580-022-00566-8. 36596869 10.1038/s41580-022-00566-8 PMC10213152

[19] Yao RW, Wang Y, Chen LL. Cellular functions of long noncoding RNAs. Nature Cell Biology. 2019. 10.1038/s41556-019-0311-8. 31048766 10.1038/s41556-019-0311-8

[20] Chodurska B, Kunej T. Long non-coding RNAs in humans: Classification, genomic organization and function. Non-coding RNA Research. 2025. 10.1016/j.ncrna.2025.01.004. 39967600 10.1016/j.ncrna.2025.01.004 PMC11833636

[21] Statello L, Guo CJ, Chen LL, Huarte M. Gene regulation by long non-coding RNAs and its biological functions. Nature Reviews Molecular Cell Biology. 2021(2). 10.1038/s41580-020-00315-9. 10.1038/s41580-020-00315-9 PMC7754182 33353982

[22] Wiener D, Schwartz S. The epitranscriptome beyond m6A. Nature Reviews Genetics. 2020;22(2):119–31. 10.1038/s41576-020-00295-8. 33188361 10.1038/s41576-020-00295-8

[23] Friedman B, Cronstein B. Methotrexate mechanism in treatment of rheumatoid arthritis. Joint, bone, spine : revue du rhumatisme. 2018;86(3):301. 10.1016/J.JBSPIN.2018.07.004. 10.1016/j.jbspin.2018.07.004 PMC6360124 30081197

[24] Di Matteo A, Bathon JM, Emery P. Rheumatoid arthritis. Lancet. 2023;402(10416):2019–33. 10.1016/S0140-6736(23)01525-8. 38240831 10.1016/S0140-6736(23)01525-8

[25] Gao Y, Zhang Y, Liu X. Rheumatoid arthritis: pathogenesis and therapeutic advances. MedComm. 2024;5(3):e509. 10.1002/MCO2.509. 38469546 10.1002/mco2.509 PMC10925489

[26] Peña-Peña M, González-Ramírez J, Bermúdez-Benítez E, Sánchez-Gloria JL, Amezcua-Guerra LM, Tavera-Alonso C, et al. Regulation of lncRNA NUTM2A-AS1 and CCR3 in the Clinical Response to a Plant-Based Diet in Rheumatoid Arthritis: A Pilot Study. Nutrients. 2025. 10.3390/nu17111752. 40507021 10.3390/nu17111752 PMC12158175

[27] Smolen JS, Aletaha D, Barton A, Burmester GR, Emery P, Firestein GS, et al. Rheumatoid arthritis. Nat Rev Dis Primers. 2018. 10.1038/nrdp.2018.1. 29417936 10.1038/nrdp.2018.1

[28] Devarakonda S, Morgensztern D, Govindan R, Lancet Publishing Group. Genomic alterations in lung adenocarcinoma. Lancet Oncol. 2015. 10.1016/S1470-2045(15)00077-7. 26149886 10.1016/S1470-2045(15)00077-7

[29] Relli V, Trerotola M, Guerra E, Alberti S. Distinct lung cancer subtypes associate to distinct drivers of tumor progression. Oncotarget. 2018;9(85):35528. 10.18632/ONCOTARGET.26217. 30473748 10.18632/oncotarget.26217 PMC6238974

[30] Lee HJ, Lee CH, Jeong YJ, Chung DH, Goo JM, Park CM, et al. IASLC/ATS/ERS international multidisciplinary classification of lung adenocarcinoma: novel concepts and radiologic implications. J Thorac Imaging. 2012;27(6):340–53. 10.1097/RTI.0B013E3182688D62. 23086014 10.1097/RTI.0b013e3182688d62

[31] Sung H, Ferlay J, Siegel RL, Laversanne M, Soerjomataram I, Jemal A, et al. Global cancer statistics 2020: GLOBOCAN estimates of incidence and mortality worldwide for 36 cancers in 185 countries. CA Cancer J Clin. 2021;71(3):209–49. 10.3322/CAAC.21660. 33538338 10.3322/caac.21660

[32] Hutchinson BD, Shroff GS, Truong MT, Ko JP. Spectrum of lung adenocarcinoma. Semin Ultrasound CT MRI. 2019;40(3):255–64. 10.1053/j.sult.2018.11.009. 10.1053/j.sult.2018.11.009 31200873

[33] Seguin L, Durandy M, Feral CC. Lung adenocarcinoma tumor origin: a guide for personalized medicine. Cancers. 2022. 10.3390/cancers14071759. 35406531 10.3390/cancers14071759 PMC8996976

[34] Zhuang J, Chen Z, Chen Z, Chen J, Liu M, Xu X, et al. Construction of an immune-related lncRNA signature pair for predicting oncologic outcomes and the sensitivity of immunosuppressor in treatment of lung adenocarcinoma. Respir Res. 2022. 10.1186/s12931-022-02043-4. 35562727 10.1186/s12931-022-02043-4 PMC9101821

[35] Liu ZY, Tang JM, Yang MQ, Yang ZH, Xia JZ. The role of LncRNA-mediated autophagy in cancer progression. Front Cell Dev Biol. 2024;12:1348894. 10.3389/FCELL.2024.1348894/FULL. 38933333 10.3389/fcell.2024.1348894 PMC11199412

[36] Bray F, Laversanne M, Hyuna S, Ferlay J, Siegel RL, et al. Global cancer statistics 2022: GLOBOCAN estimates of incidence and mortality worldwide for 36 cancers in 185 countries. CA Cancer J Clin. 2024;74(3):229–63. 10.3322/CAAC.21834. 38572751 10.3322/caac.21834

[37] Hossain MS, Karuniawati H, Jairoun AA, Urbi Z, Ooi DJ, John A, et al. Colorectal Cancer: A Review of Carcinogenesis, Global Epidemiology, Current Challenges, Risk Factors, Preventive and Treatment Strategies. Cancers. 2022;14(7):1732. 10.3390/CANCERS14071732. 35406504 10.3390/cancers14071732 PMC8996939

[38] Wei L, Wang X, Lv L, Zheng Y, Zhang N, Yang M. The emerging role of noncoding RNAs in colorectal cancer chemoresistance. Cell Oncol. 2019;42(6):757–68. 10.1007/S13402-019-00466-8/FIGURES/1. 10.1007/s13402-019-00466-8 PMC12994294 31359293

[39] Vodenkova S, Buchler T, Cervena K, Veskrnova V, Vodicka P, Vymetalkova V. 5-fluorouracil and other fluoropyrimidines in colorectal cancer: past, present and future. Pharmacol Ther. 2020;206:107447. 10.1016/J.PHARMTHERA.2019.107447. 31756363 10.1016/j.pharmthera.2019.107447

[40] Wu K, Liang WC, Feng L, Pang J, Waye MMY, Zhang JF, et al. H19 mediates methotrexate resistance in colorectal cancer through activating Wnt/β-catenin pathway. Exp Cell Res. 2017;350(2):312–7. 10.1016/J.YEXCR.2016.12.003. (**PubMed PMID: 27919747**). 27919747 10.1016/j.yexcr.2016.12.003

[41] Ji Z, Shen J, Lan Y, Yi Q, Liu H. Targeting signaling pathways in osteosarcoma: mechanisms and clinical studies. MedComm (Beijing). 2023;4(4):e308. 10.1002/MCO2.308. 10.1002/mco2.308 PMC10333890 37441462

[42] Isakoff MS, Bielack SS, Meltzer P, Gorlick R. Osteosarcoma: current treatment and a collaborative pathway to success. J Clin Oncol. 2015;33(27):3029–35. 10.1200/JCO.2014.59.4895/ASSET/9D5BD968-520F-4A0E-8934-DA8ADC9C1617/ASSETS/GRAPHIC/ZLJ02715-5266-T01.JPEG. 26304877 10.1200/JCO.2014.59.4895 PMC4979196

[43] Mirabello L, Troisi RJ, Savage SA. International osteosarcoma incidence patterns in children and adolescents, middle ages and elderly persons. Int J Cancer. 2009;125(1):229–34. 10.1002/IJC.24320. 19330840 10.1002/ijc.24320 PMC3048853

[44] Whelan JS, Davis LE. Osteosarcoma, chondrosarcoma, and chordoma. J Clin Oncol. 2018;36(2):188–93. 10.1200/JCO.2017.75.1743. 29220289 10.1200/JCO.2017.75.1743

[45] Kansara M, Teng MW, Smyth MJ, Thomas DM. Translational biology of osteosarcoma. Nat Rev Cancer. 2014;11(11):722–35. 10.1038/nrc3838. 10.1038/nrc3838 25319867

[46] Levêque D, Santucci R, Gourieux B, Herbrecht R. Pharmacokinetic drug-drug interactions with methotrexate in oncology. Expert Rev Clin Pharmacol. 2011;6(6):743–50. 10.1586/ECP.11.57. 10.1586/ecp.11.57 22111860

[47] Drago P, Bookey N, Leung KY, Henry M, Meleady P, Greene NDE, et al. DHFR2 RNA directly regulates dihydrofolate reductase and its expression level impacts folate one carbon metabolism. FASEB J. 2025;(4):e70391. 10.1096/FJ.202401039RR. 39957677 10.1096/fj.202401039RR PMC11831416

[48] McLendon R, Friedman A, Bigner D, Van Meir EG, Brat DJ, Mastrogianakis GM, et al. Comprehensive genomic characterization defines human glioblastoma genes and core pathways. Nature. 2008Oct 23;455(7216):1061–8. 10.1038/NATURE07385. (**PubMed PMID: 18772890**). 10.1038/nature07385 PMC2671642 18772890

[49] Louis DN, Perry A, Wesseling P, Brat DJ, Cree IA, Figarella-Branger D, et al. The 2021 WHO classification of tumors of the central nervous system: a summary. Neuro Oncol. 2021;8(8):1231–51. 10.1093/NEUONC/NOAB106. 10.1093/neuonc/noab106 PMC8328013 34185076

[50] Grochans S, Cybulska AM, Simińska D, Korbecki J, Kojder K, Chlubek D, et al. Epidemiology of glioblastoma multiforme–literature review. Cancers (Basel). 2022;(10):2412. 10.3390/CANCERS14102412. 35626018 10.3390/cancers14102412 PMC9139611

[51] Dang Y, Zhou D, Du X, Zhao H, Lee CH, Yang J, et al. Molecular mechanism of substrate recognition by folate transporter SLC19A1. Cell Discov. 2022;1(1):1–11. 10.1038/S41421-022-00508-W. 10.1038/s41421-022-00508-w PMC9794768 36575193

[52] Xie P, Yan H, Gao Y, Li X, Zhou DB, Liu ZQ. Construction of m6A-related lncRNA prognostic signature model and immunomodulatory effect in glioblastoma multiforme. Front Oncol. 2022. 10.3389/fonc.2022.920926. 35719945 10.3389/fonc.2022.920926 PMC9201336

[53] Liu CQ, Xia QD, Sun JX, Xu JZ, Lu JL, Liu Z, et al. Identification and validation of a twelve immune infiltration-related lncRNA prognostic signature for bladder cancer. Aging. 2022;14(3):1492–507. 10.18632/AGING.203889. 35165206 10.18632/aging.203889 PMC8876923

[54] Liu J, Liu Y, Gao F, Zhang J, Pan J, Liu Y, et al. Comprehensive study of a novel immune-related lncRNA for prognosis and drug treatment of cervical squamous cell carcinoma. Am J Transl Res [Internet]. 2021. Report. Available from: http://www.gen- PMC8581925 34786106

[55] Liu X, Cheng W, Li H, Song Y. Identification and validation of cuproptosis-related LncRNA signatures as a novel prognostic model for head and neck squamous cell cancer. Cancer Cell Int. 2022. 10.1186/s12935-022-02762-0. 36369058 10.1186/s12935-022-02762-0 PMC9652850

[56] Li C, Liang G, Yang S, Sui J, Yao W, Shen X, et al. Dysregulated lncRNA-UCA1 contributes to the progression of gastric cancer through regulation of the PI3K-Akt-mTOR signaling pathway. Oncotarget. 2017;8(55):93476–91. 10.18632/ONCOTARGET.19281. 29212166 10.18632/oncotarget.19281 PMC5706812

[57] Ounzain S, Pezzuto I, Micheletti R, Burdet F, Sheta R, Nemir M, et al. Functional importance of cardiac enhancer-associated noncoding RNAs in heart development and disease. J Mol Cell Cardiol. 2014;76:55. 10.1016/J.YJMCC.2014.08.009. 25149110 10.1016/j.yjmcc.2014.08.009 PMC4445080

[58] Xu R, Sun Y, Tian F, Zhao M. LncRNA NEAT1 sponges miR-214-3p to promote osteoblast differentiation through regulating the PI3K/AKT/mTOR pathway in aortic valve calcification. Scientific Reports 2025 15:1. 2025;15(1):13665. 10.1038/s41598-025-98578-9. 10.1038/s41598-025-98578-9 PMC12012154 40258988

[59] Sherif IO, Al-Shaalan NH, Sabry D. *Ginkgo biloba* extract alleviates methotrexate-induced renal injury: new impact on pi3k/akt/mtor signaling and malat1 expression. Biomolecules. 2019. 10.3390/biom9110691. 31684190 10.3390/biom9110691 PMC6920877

[60] Chen Y, Li Z, Chen X, Zhang S. Long non-coding RNAs: from disease code to drug role. Acta Pharm Sin B. 2021;11(2):340–54. 10.1016/J.APSB.2020.10.001. 33643816 10.1016/j.apsb.2020.10.001 PMC7893121

[61] Wang Y, Fang Z, Hong M, Yang D, Xie W. Long-noncoding RNAs (lncRNAs) in drug metabolism and disposition, implications in cancer chemo-resistance. Acta Pharm Sin B. 2020;10(1):105–12. 10.1016/J.APSB.2019.09.011. 31993309 10.1016/j.apsb.2019.09.011 PMC6976993

